# Supplementary material for: An Emerging Human Parechovirus Type 5 Causing Sepsis-Like Illness in Infants in Australia
Source: Viruses. 2019 Oct 3;11(10):913. doi: 10.3390/v11100913 (PMC6832754; doi:10.3390/v11100913)
Supplement: Supplementary file 1 [file viruses-11-00913-s001.pdf]

Supplementary Materials

An emerging human parechovirus type 5 causing sepsis-like illness in infants in Australia

Anthony Chamings[1,2], Kwee Chin Liew[3,4], Emily Reid[3], Eugene Athan[1,2,3], Amy Raditsis[2,3], Peter Vuillermin[2,3], Yano Yoga[5], Leon Caly[5], Julian Druce[5] & Soren Alexandersen[1,2,3*]

[1]Geelong Center for Emerging Infectious Diseases, Geelong, Victoria 3220, Australia

[2]Deakin University, School of Medicine, Geelong, Victoria 3220, Australia

[3]Barwon Health, University Hospital Geelong, Geelong, Victoria 3220, Australia

[4]Australian Clinical Labs, Geelong Laboratory, Geelong, Victoria 3220, Australia

[5]Victorian Infectious Diseases Reference Laboratory (VIDRL), Doherty Institute, Melbourne, Victoria, 3000, Australia

[*]Corresponding Author: Soren Alexandersen, Geelong Centre for Emerging Infectious Diseases, Health Education and Research Building Level 3, 285-299 Ryrie Street, Geelong, Victoria 3220, Australia

E-mail: soren.alexandersen@deakin.edu.au

Phone: +61 (0)342159635

Running Title

Sepsis-like illness in infants caused by human parechovirus type 5

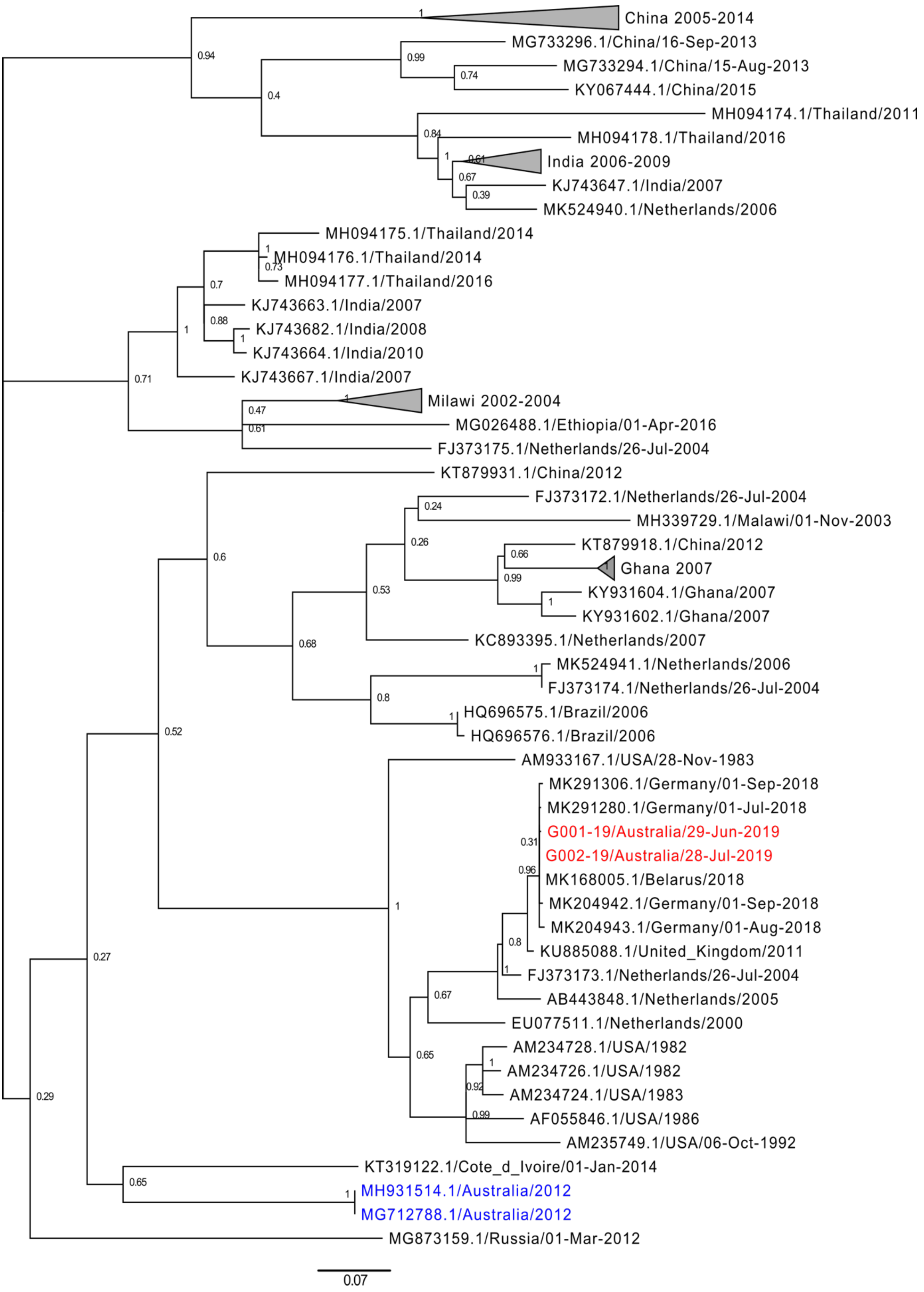

Supplementary figure 1. Maximum likelihood tree of 567nt of all HPeV5 VP1 sequences available on GenBank, August 2019. The tree was generated in Mega X using the GTR+G+I model. Branch support was determined with 1000 bootstrap replicates and is shown on the branch nodes. The HPeV5

sequences described in this paper are in red. Other Australian HPeV5 VP1 sequences available on GenBank are in blue.
